# Interplay between Genome, Metabolome and Microbiome in Colorectal Cancer

**DOI:** 10.3390/cancers13246216

**Published:** 2021-12-10

**Authors:** Koldo Garcia-Etxebarria, Marc Clos-Garcia, Oiana Telleria, Beatriz Nafría, Cristina Alonso, Marta Iruarrizaga-Lejarreta, Andre Franke, Anais Crespo, Agueda Iglesias, Joaquín Cubiella, Luis Bujanda, Juan Manuel Falcón-Pérez

**Affiliations:** 1 Grupo de Genética Gastrointestinal, Biodonostia, 20014 San Sebastián, Spain; 2 Centro de Investigación Biomédica en Red de Enfermedades Hepáticas y Digestivas (CIBERehd), 08036 Barcelona, Spain; joaquin.cubiella.fernandez@sergas.es (J.C.); luis.bujandafernandezdepierola@osakidetza.eus (L.B.); jfalcon@cicbiogune.es (J.M.F.-P.); 3 Exosomes Laboratory, Centro de Investigación Cooperativa en Biociencias (CIC bioGUNE), 48160 Derio, Spain; marc.clos.garcia@sund.ku.dk (M.C.-G.); oiana.telleria@biodonostia.org (O.T.); 4 Grupo de Enfermedades Gastrointestinales, Biodonostia, Universidad del País Vasco (UPV/EHU), 20014 San Sebastián, Spain; beatriz.nafriajimenez@osakidetza.eus; 5 OWL Metabolomics, Bizkaia Technology Park, 48160 Derio, Spain; calonso@owlmetabolomics.com (C.A.); miruarrizaga@owlmetabolomics.com (M.I.-L.); 6 Institute of Clinical Molecular Biology, Christian-Albrechts-University of Kiel, 24105 Kiel, Germany; a.franke@ikmb.uni-kiel.de; 7 Department of Gastroenterology, Instituto de Investigación Sanitario Galicia Sur, Complexo Hospitalario Universitario de Ourense, 32005 Ourense, Spain; anais.crespo.lois@sergas.es (A.C.); agueda.iglesias.gomez@sergas.es (A.I.); 8 Basque Foundation for Sciences, Ikerbasque, 48013 Bilbao, Spain; 9 Metabolomics Platform, Centro de Investigación Cooperativa en Biociencias (CIC bioGUNE), 48160 Derio, Spain

**Keywords:** colorectal cancer, adenoma, metabolome, microbiome, host genome

## Abstract

**Simple Summary:**

The development of colorectal cancer (CRC) is influenced by the environment, genetics and microbiota. Microbiome and metabolome analyses allowed for the finding of factors and markers associated with adenoma and CRC risk, but the interaction of host genomics with those omic layers remains unclear. Thus, our aim is to add host genome information to find new factors and markers associated with adenoma and CRC risk or to propose biological mechanisms involved in the risk. We found interactions between different omic layers that could be biologically relevant, and the three layers gave complementary information to predict adenoma and CRC risk. Our findings will help to find new markers for adenoma and CRC risk and to analyze biological mechanisms involved in adenoma and CRC development.

**Abstract:**

Background: Colorectal cancer (CRC), a major health concern, is developed depending on environmental, genetic and microbial factors. The microbiome and metabolome have been analyzed to study their role in CRC. However, the interplay of host genetics with those layers in CRC remains unclear. Methods: 120 individuals were sequenced and association analyses were carried out for adenoma and CRC risk, and for selected components of the microbiome and metabolome. The epistasis between genes located in cholesterol pathways was analyzed; modifiable risk factors were studied using Mendelian randomization; and the three omic layers were used to integrate their data and to build risk prediction models. Results: We detected genetic variants that were associated to components of metabolome or microbiome and adenoma or CRC risk (e.g., in *LINC01605*, *PROKR2* and *CCSER1* genes). In addition, we found interactions between genes of cholesterol metabolism, and HDL cholesterol levels affected adenoma (*p* = 0.0448) and CRC (*p* = 0.0148) risk. The combination of the three omic layers to build risk prediction models reached high AUC values (>0.91). Conclusions: The use of the three omic layers allowed for the finding of biological mechanisms related to the development of adenoma and CRC, and each layer provided complementary information to build risk prediction models.

## 1. Introduction

Colorectal cancer (CRC) is a major health concern that represents around 10% of the cancer incidence in the world [1], and it is the second cause of cancer death in developed countries [2]. CRC can be developed sporadically or as consequence of inflammatory processes [3], and its development is influenced by environmental factors, such as physical activity or diet [4], genetic factors and the microbiome composition [5]. Due to the burden that CRC entails, different screening strategies and diagnostic biomarkers have been developed to do an early detection of CRC [6].

As mentioned, genetic factors are involved in CRC risk and, therefore, they can be appropriate biomarkers. The study of genetic markers and their role in CRC risk have been carried out through the analysis of specific genetic variants [7,8,9] and genome-wide association studies (GWAS) [10]. In addition, the effect of lipid level on colorectal polyps [11] and CRC [12], as well as the effect of other modifiable risk factors [13], have been detected through genetic Mendelian randomization analyses. Finally, based on the information of those analyses, polygenic risk scores (PRS) have been developed to predict the risk of CRC based on the carriership of risk genetic variants, among other factors [14,15].

In the search of non-invasive biomarkers, in our previous works, we have analyzed the state, contribution and interaction of metabolome [16] and microbiome [17] to CRC risk. We found that six metabolites, among others, metabolites of cholesterol metabolism, and 16 bacteria genera that can be identified from fecal samples were able to discriminate cases from healthy controls [16,17]. In addition, we found alterations in the presence of the phyla Bacteroidete, Firmicutes and Fusobacteria in CRC or adenoma patients [17]. It has been suggested that the state of one omic layer could affect the state of other layers and their interplay could determine the final phenotype [18], e.g., the host genomic layer can influence the microbiome layer [19]. In addition, it has been proposed that the enterocytes are exposed to the metabolites produced by the diet, intestinal secretions and the digestion done by microbes; and the enterocytes can interact directly with microbes [20,21], but the effect of the host genetics in those interactions remains unclear, since, as far as we know, analyses of genome, metabolome and microbiome and their interplay are scarce in CRC, and limited to animal models [22].

Thus, our aim in this work is to add the information from host genomic layer to our previous results in metabolomic and microbiome layers to further characterize their interplay and effect in adenoma an CRC risk and development.

## 2. Materials and Methods

### 2.1. Samples

The patients included in this study were recruited from the COLONPREDICT study [23] and from the Biobank of the Instituto de Investigación Sanitaria Galicia Sur (http://www.iisgaliciasur.es/home/biobank-iisgs/?lang=en accessed on 3 December 2021). Those patients were referred to a colonoscopy due to gastrointestinal symptomatology; and patients with the following characteristics were excluded: age under 18, pregnancy, patients with previous history of colonic disease, patients requiring hospital admission, patients whose symptoms had ceased within three months of evaluation, and patients who declined to participate after reading the informed consent form. This study was approved by the Clinical Research Ethics Committee of Galicia (Code 2011/038).

The samples and the clinical information were obtained from COLONPREDICT study and from the biobank. Patients were classified in three categories: normal, advanced adenoma (≥10 mm, villous histology or high-grade dysplasia) and colorectal cancer (invasive adenocarcinoma). Unfortunately, information regarding the CRC stage or the characteristics of the advanced adenomas was not available in the samples obtained from the biobank. Thus, this information could not be incorporated into the analyses.

### 2.2. Data Generation

The procedure to obtain metabolome and microbiome was described elsewhere [16,17]. Briefly, metabolomics analysis was carried out analyzing chloroform/methanol extracts using a UHPLC−time-of-flight (TOF)-MS-based platform. In the case of microbiome, variable regions V1 and V2 of the 16S rRNA gene were sequenced using Illumina MiSeq v3 (Illumina Inc., San Diego, CA, USA). Both omic layers were retrieved from matched fecal samples. Fecal hemoglobin (f-Hb) concentration was determined within the COLONPREDICT study in all patients using the automated OC-SENSOR MICRO analyser (Eiken Chemical Co., Ltd., Tokyo, Japan).

DNA of the individuals included in this study was extracted from blood. The Illumina Global Screening Array on Illumina iScan high-throughput screening system was used to genotype the individuals in the Institute of Clinical Molecular Biology (Kiel, Germany). The raw intensities were then analyzed using Illumina GenomeStudio software and GenCall algorithm was applied to call the alleles.

Once genotyped, samples and markers were analyzed using the quality control (QC) criteria usually applied in our group: exclusion of samples with ≥15% missing rates; exclusion of markers with non-called alleles; exclusion of markers with missing call rates > 0.05; exclusion of samples with ≥5% missing rates; exclusion of related samples (PI-HAT > 0.1875); exclusion of samples whose genotyped sex could not be determined; exclusion of samples with high heterozygosity rate (more than three times SD from the mean); only autosomal SNPs were kept; removal of markers with Hardy-Weinberg equilibrium *p* < 1 × 10^−5^; removal of markers whose P of difference in missingness between cases and control was <1 × 10^−5^; and removal of samples which were outliers, identified using principal component analysis (deviation of more than 6 times interquartile range).

The QCed samples were imputed using the Sanger Imputation service. Haplotype Reference Consortium release 1.1 was used as reference panel and the pipeline EAGLE2 + PBWT was used to do the imputation. From the imputation we retrieved markers with INFO score ≥ 0.80 and biallelic markers.

After genotyping, quality control and imputation, 5,411,755 SNPs from 120 individuals (39 patients with adenomas, 41 colorectal cancer cases, and 40 controls) were kept. From those individuals, 104 (36 adenomas, 34 colorectal cancer and 34 controls) had available information for metabolome and microbiome.

### 2.3. Analyses

In genetic association analyses of adenoma and CRC, all genotyped individuals were used, regardless of the availability of metabolome and microbiome information. The analyses of adenoma vs. controls (N = 79), CRC vs. controls (N = 81), CRC vs. adenoma (N = 80) and CRC + adenoma vs. controls (N = 120) were carried out using logistic regression implemented in Plink [24], adjusting by age, sex and first 4 principal components. 

In addition, linear regression implemented in Plink [24], adjusting by age, sex and the first four principal components was used to analyze the proportional abundance of Bacteroidetes (N = 104) and Firmicutes (N = 104); values of the Shannon index (N = 104); and the levels of ChoE(18:1) (N = 116), ChoE(18:2) (N = 116), ChoE(20:4) (N = 116), PE(16:0/18:1) (N = 116), SM(42:3) (N = 116) and TG(54:1) (N = 116); and logistic regression, adjusting by age, sex and the first four principal components, to analyze the presence of Fusobacteria (N = 104).

Analyses of epistasis of SNPs located in genes of interest were carried out using Plink [24]. In the case of “cholesterol esters metabolism” pathway, 1860 available SNPs were interrogated for interactions between them; and in the case of “Glycerophospholipid, Sphingolipid metabolism, Glycosylphosphatidylinositol(GPI)-anchor biosynthesis” pathway, 1925 SNPs. 

For carrying out Mendelian randomization (MR) analyses, TwoSampleMR [25] and gsmr [26] packages from R language were used [27]. First, the instruments (SNPs) for the traits of interest that are known to affect CRC (BMI, cholesterol, triglycerides, selenium, iron, vitamin B12, metabolism, body fat percentage, waist circumference, IL6 receptor and height [13]) available in MRC-IEU (https://gwas.mrcieu.ac.uk accessed on 15 April 2021) were retrieved through TwoSampleMR [25], and the analysis was carried out if 10 or more instruments were available. Then HEIDI outlier analysis was used to discard heterogenous instruments. With those instruments we carried out the MR analysis using MR Egger, as done in CRC previously [13]. The analysis was done for each comparison (adenoma vs. controls, CRC vs. controls, CRC vs. adenoma, and CRC + adenoma vs. controls).

To integrate the data from the three omic layers studied in this work (genome, metabolome and microbiome) MOFA2 package [28,29] of R language [27] was used. In genome, SNPs that where genotyped and have a MAF > 0.05 were included, in total 98,966 SNPs; in metabolome, 90 metabolites were included; and in microbiome 120 microbial genera were included, after removing the less variable genera using nearZeroVar function included in the package “caret” (https://CRAN.R-project.org/package=caret accessed on 28 June 2021) of R language. The analysis in MOFA2 was carried out using default settings.

Polygenic risk scores (PRS) were calculated using PRSice software [30]. As base summary statistics, 397 SNPs associated to CRC retrieved from GWAS Catalog [31] were used; the additive model was tested; and the analysis was adjusted by age, sex and the first four principal components. The analyses were performed in adenoma vs. controls, CRC vs. controls, CRC vs. adenoma, and CRC + adenoma vs. controls.

To build and test predictive models using metabolome (ChoE(18:1), ChoE(18:2), ChoE(20:4), PE(16:0/18:1), SM(42:3) and TG(54:1) levels), microbiome (*Fusobacterium*, *Gemella*, *Blautia*, *Butyrivibrio*, *Clostridium*, *Coprococcus*, *Dorea*, *Peptococcus*, *Peptostreptococcus*, *Staphylococcus*, *Streptococcus*, *Parvimonas* and *Selenomonas* genera abundance) and genome (PRS and PC1-PC4) data, a logistic regression was used in R language [27] for each comparison (adenoma vs. controls, CRC vs. controls, CRC vs. adenoma, and CRC + adenoma vs. controls). The models tested were sex, age, metabolome and microbiome (Model1); age, sex, metabolome, microbiome and fecal hemoglobin (Model 1 + f-Hb); age, sex and genetics (Model 2); age, sex, genetics and fecal hemoglobin (Model 2 + f-Hb); sex, age, metabolome, microbiome and genetics (Model 1 + Model 2); and age, sex, metabolome, microbiome, genetics and fecal hemoglobin (Model 1 + Model 2 + f-Hb). To calculate the performance of the model, the sensitivity, the specificity and the area under de curve pROC package of R language [27] was used.

## 3. Results

### 3.1. Genetic Associations of Adenoma and Colorectal Cancer

In the genetic analyses of adenoma vs. healthy controls (AD vs. C), colorectal cancer vs. healthy controls (CRC vs. C), colorectal cancer vs. adenoma (CRC vs. AD) or adenomas and colorectal cancer vs. controls (CRC + AD vs. C) there was not any genome-wide significant SNP (*p* < 5 × 10^−8^). The best SNP in each analysis were rs7178030 (*p* = 9.76 × 10^−5^), rs11125152 (*p* = 0.0001), rs33603 (*p* = 8.59 × 10^−5^) and rs6510888 (*p* = 1.58 × 10^−5^), respectively.

Among the SNPs previously associated with CRC (Supplementary Table S1), 2 of them had nominal p-value (*p* < 0.05), in AD vs. C, 3 in CRC vs. C, 1 in CRC vs. AD and 1 in CRC + AD vs. C. In the case of SNPs associated with CRC in Spanish cohorts, none of them were significant.

When the association of SNPs located in genes of interest were inspected significant results (*p* < 0.05) were found. In the case of genes involved in cholesterol esters metabolism pathway, in AD vs. C 7 genes had SNPs with *p* < 0.05 value, 6 in CRC vs. C, 7 in CRC vs. AD and 7 CRC + AD vs. C (Table 1). In the case of genes involved in Glycerophospholipid, Sphingolipid metabolism, Glycosylphosphatidylinositol(GPI)-anchor biosynthesis, in AD vs. C 8 genes had SNPs with *p* < 0.05 value, 10 in CRC vs. C, 6 in CRC vs. AD and 9 CRC + AD vs. C (Table 2).

**Figure 1:**
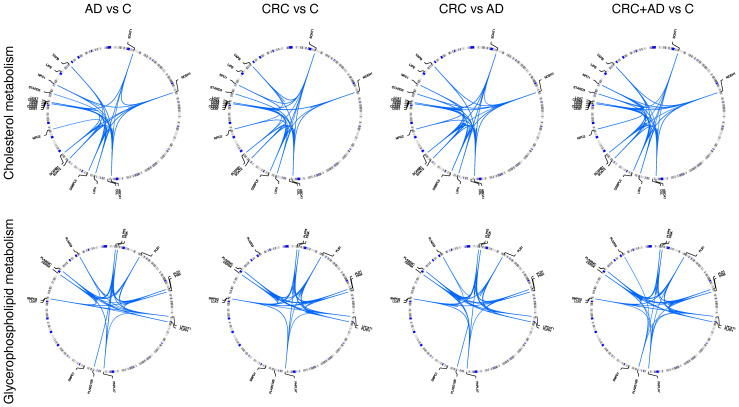
Epistatic interaction between SNPs of genes of cholesterol esters metabolism and Glycerophospholipid, Sphingolipid metabolism, Glycosylphosphatidylinositol(GPI)-anchor biosynthesis pathways.

Moreover, epistatic interactions between the genes of those pathways were analyzed (Figure 1). The landscape of the interaction between genes was slightly different in each analysis, for example, the connections between *SOAT1* and *SCARB1* was different in CRC vs. C and in CRC vs. AD. On the whole, the number of interactions was higher in AD + CRC vs. C than in the rest of comparisons and all genes carried SNPs that interact with SNPs of other genes, with the exception of *CEL* and *LIPE* genes in cholesterol esters metabolism pathway; and *SMPD1* gene in Glycerophospholipid, Sphingolipid metabolism, Glycosylphosphatidylinositol(GPI)-anchor biosynthesis, as well as *PLA2G3* and *PLA2G12B* genes but only in CRC vs. C (Figure 1).

### 3.2. Genetic Associations of Selected Microbiome and Metabolome Traits

Among the 34 suggestive signals found in the 4 traits related to the microbiome (abundance of Bacteroidetes 9 suggestive *loci*, abundance of Firmicutes 8, and values of Shannon index 17; while there was not any suggestive signal in the presence of Fusobacteria) there were six signals that showed nominal significance in adenoma or CRC (Table 3): rs2470641, which decreases the abundance of Bacteroidetes, decreases the risk of CRC (*p* = 0.049, OR = 0.3); four SNPs which were involved in the abundance of Firmicutes, were associated with higher AD risk (rs9538330 and rs7166734), or they had different frequencies in CRC and AD (rs2662642 and rs13280938); and rs73523611, which increases the Shannon diversity index, was significant in AD vs. C (*p* = 0.044, OR = 3.5).

In the case of the metabolites analyzed, among the 168 suggestive signals (25 loci in ChoE(18:1), 3 in ChoE(18:2), 12 in ChoE(20:4), 41 in PE(16:0/18:1), 12 in SM(42:3), 58 in TG(54:1)), 17 signals showed an association with adenoma or CRC (Table 3). Three SNPs involved in the levels of ChoE(18:1) had *p* < 0.05 in CRC vs. C or CRC + AD vs. C analyses. In the case of ChoE(18:2) rs62249239 had *p* < 0.05 in CRC vs. AD (*p* = 0.036; OR = 9.8). Four SNPs that affect PE(16:0/18:1) levels had *p* < 0.05 in AD vs. C, CRC vs. C or CRC + AD vs. C analyses. Six SNPs that affect SM(42:3) levels had *p* < 0.05 in CRC vs. C, CRC vs. AD or CRC + AD vs. C analyses. In the case of TG(54:1), rs117956865 had an association in CRC vs. AD (*p* = 0.039, OR = 12.1). Among those SNPs, rs6085078 was associated with the increase of ChoE(18:1) and SM(42:3) levels and it was associated in CRC vs. C analysis (*p* = 0.021, OR = 2.9) and CRC + AD vs. C (*p* = 0.037, OR = 2.0).

### 3.3. Mendelian Randomization

**Figure 2:**
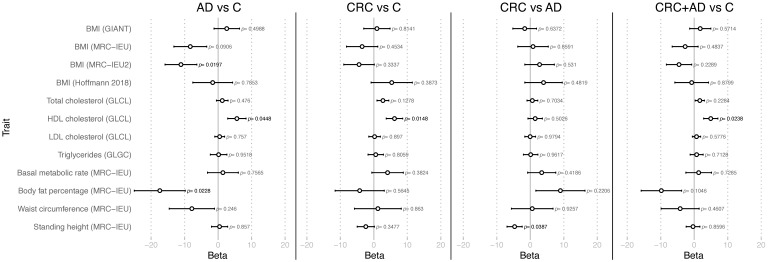
Mendelian Randomization analyses in adenoma and colorectal cancer. AD vs. C, adenoma vs. healthy controls; CRC vs. C, colorectal cancer vs. healthy controls; CRC vs. AD, colorectal cancer vs. adenoma; CRC + AD vs. C, adenomas and colorectal cancer vs. controls. Between parenthesis, the source of the SNPs used as exposure in the analysis.

Traits that have been previously described to affect CRC were used in a Mendelian randomization analysis to measure their effect in the adenoma and CRC traits of our cohort (Figure 2). In the case of AD vs. C, BMI (P = 0.0197) and body fat percentage (*p* = 0.0228) affected negatively, while HDL cholesterol affected positively (*p* = 0.0448); in CRC vs. C HDL cholesterol affected positively (*p* = 0.0148); in CRC vs. AD standing height affected negatively (*p* = 0.0387); and in CRC + AD vs. C HDL cholesterol affected positively (*p* = 0.0238). 

### 3.4. Multiomic Integration

**Figure 3:**
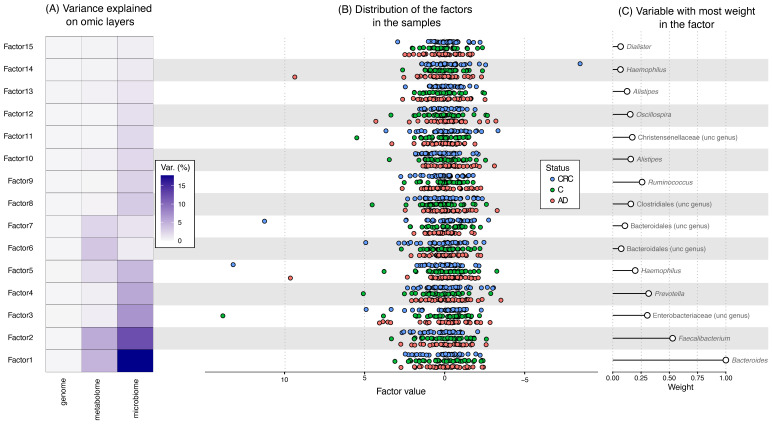
Factors derived from the integration of genome, metabolome and microbiome data. (**A**) The variance explained by each omic layer in each Factor. (**B**) the distribution of the values of the factors in each sample. CRC, colorectal cancer; C, healthy controls; AD, adenoma. (**C**) The variable with the most weight in the Factor. “Unc genus”, genus that was not classified, the identified lowest level is showed.

The integration of the data from the three omic layers analyzed in this work showed that microbiome, represented by microbial genera, was the layer which more variance explained (Figure 3). In addition, the metabolome explained part of the variation, especially in Factors 1 and 2, while the host genome did not explain the variation. Except for Factor 1 and 2 for metabolome and microbiome, there was not any shared covariance among the different omic layers.

The distribution of the factors in the samples showed that some samples had extreme values, while most of the samples had a similar distribution of the factors regardless their status. Although some clustering can be identified (some AD samples had higher values of Factor 3 and CRC samples in Factor 6), there was not any clear difference between the different status (Figure 3).

In all the Factors the variable with most weight come from the microbiome layer (Figure 3): *Bacteroides* for Factor 1 and *Faecalibacterium* for Factor 2 were the factors with highest weight.

### 3.5. Polygenic Risk Scores and Predictive Model

Polygenic risk scores were computed using the information of SNPs previously associated to CRC and optimized for each analysis: in AD vs. CRC 147 SNPs were used, in CRC vs. C 191 SNPs, in CRC vs. AD 69 SNPs, and in CRC + AD vs. C 147 SNPs. Based on those SNPs the PRS for each individual and analysis was calculated.

Those PRS scores, as well as the genetic distance of the individuals (reflected in the first 4 PC components), were applied to build a predictive model. Compared with the previous model built by our group using metabolome and microbiome data, the performance of the use of genetic information was not better in any of the four comparisons (Table 4). The inclusion of fecal hemoglobin (f-Hb) test as predictive value improved the performance of genetic analyses, and the combination of genetics and f-Hb improved the combination of microbiome and metabolome in CRC + AD vs. control (Table 4). Finally, the combination of metabolome, microbiome and genetic data improved the predictive model in the four comparisons (Table 4).

## 4. Discussion

In the present study we have taken advantage of adding host genetic information to the omic layers that we studied previously, namely, the metabolome [16] and microbiome [17]. As far as we know, the interplay between those layers in CRC have been analyzed in animal models [22], but not in humans. On that study two mice strains were compared, since they showed differences in the number of intestinal adenomas, although one strain was the founder of the other; and the two strains have gut microbiota from two different sources [22]. The number of adenomas was influenced by the genetic variation and microbiome, and the metabolome was useful to identify the relevant biological functions affected by genetic variation [22]. In our case, it was expected that the heterogeneity of genome and microbiome would be higher and, therefore, the results could not be completely comparable. However, in both works the multi-omic approach has been useful to find biological functions relevant for the development of adenoma or colorectal cancer.

Due to our sample size the statistical power of our analyses was limited. As a consequence, we were not able to find new significant genetic variants associated to adenoma or CRC, or to find significant results for genetic variants previously associated to CRC [7,8,9], but we were able to find connections with other layers, to measure the effect of modifiable risk factors, and to predict the risk of CRC. In addition, we could not include histopathological characteristics to our analyses due to the lack of information in some samples, and we only compare the three categories (controls, adenoma and CRC). Although some biases could be present in the statistical analyses due to the sample size, the most relevant findings were consistent with previous works.

Among the SNPs that were associated to microbiome or metabolome traits and had nominal association with adenoma or CRC, we found genetic variants in genes associated with CRC: rs13280938 is located in the long non-coding RNA *LINC01605*, which has been described as important regulator in CRC [32]; rs6085078 of *PROKR2* gene, a receptor which is associated with colorectal progression [33]; and rs17018424 of *CCSER1* gene, a gene involved in cell division defect [34], and the overexpression of a deletion in this gene results in overexpression of adenocarcinoma associated genes [35]. Thus, our approach allowed to propose biological mechanisms that could link components of different layers. Those biological mechanisms could explain the detection of the risk of developing adenoma and colorectal cancer by different omic layers and, therefore, their utility as risk biomarkers. The present results could be a basis to suggest follow-up analyses and analyses in cell- and animal-models to understand such mechanisms and the implications for adenoma and CRC risk.

In the case of modifiable risk factors, we were able to detect the effect of the levels of HDL cholesterol, body fat percentage and BMI in adenoma and CRC, results that are consistent with previous studies [11,12,13]. Thus, host genome and metabolome indicated the importance of the role of cholesterol in adenoma and CRC risk, since some relevant metabolites from the metabolome analysis were cholesterol products [16]. When we scrutinized the SNPs of genes of pathways related to cholesterol metabolism, there were some significant (*p* < 0.05) SNPs, but more interestingly, the epistatic interactions between SNPs of those genes were significant. Thus, the results observed in the metabolome layer could be an effect of interaction between genes, rather than the effect of individual genetic variants; and that cholesterol levels are a risk factor also in adenoma.

When genetic information was used to build the predictive model of risk, the genetic markers used were not as good as the model built using metabolome and microbiome [17] information. However, when the information of the three layers was combined, the performance was higher than when they were separated. That could suggest that the risk factors captured by each omic layer is slightly different and complementary. This complementarity of the omic layers is consistent with the lack of covariance we observed in the integration of the three layers.

## 5. Conclusions

In conclusion, using the information of three omic layers, we were able to detect some interactions between them to propose biological mechanisms implicated in the development of adenoma and CRC which could be interesting to analyze in follow-up studies and to build an informative model for predicting the risk to adenoma and CRC.

## Figures and Tables

**Table 1:** Association study results of significant SNPs in genes of “Cholesterol esters metabolism pathway”. AD vs. C, adenoma vs. healthy controls; CRC vs. C, colorectal cancer vs. healthy controls; CRC vs. AD, colorectal cancer vs. adenoma; CRC + AD vs. C, adenomas and colorectal cancer vs. controls. OR, odds-ratio; CI 95%, 95% of confidence interval of odds-ratio.

Gene	Analysis	SNP	*p*	OR (CI 95%)
*LIPA*	AD vs. C	rs2246833	0.019	2.9 (1.2–7.1)
*LIPA*	CRC vs. AD	rs885561	0.028	0.4 (0.2–0.9)
*NCEH1*	AD vs. C	rs17756312	0.019	12.3 (1.5–100.7)
*NCEH1*	CRC vs. C	rs522028	0.017	3.8 (1.3–11.2)
*NCEH1*	CRC vs. AD	rs630736	0.049	0.3 (0.1–1.0)
*NCEH1*	CRC + AD vs. C	rs522028	0.014	2.8 (1.2–6.2)
*CES5A*	AD vs. C	rs34266217	0.013	0.1 (0.0–0.6)
*CES5A*	CRC vs. C	rs34266217	0.016	0.2 (0.0–0.7)
*CES5A*	CRC vs. AD	rs11864688	0.003	0.1 (0.0–0.4)
*CES5A*	CRC + AD vs. C	rs34266217	0.004	0.2 (0.1–0.6)
*SOAT1*	CRC vs. C	rs61824389	0.012	5.4 (1.5–20.2)
*SOAT1*	CRC vs. AD	rs61824371	0.028	2.6 (1.1–6.0)
*ABCA1*	AD vs. C	rs4149297	0.011	0.2 (0.0–0.7)
*ABCA1*	CRC vs. C	rs2487060	0.029	9.4 (1.2–70.3)
*ABCA1*	CRC vs. AD	rs2515617	0.007	0.3 (0.1–0.7)
*ABCA1*	CRC + AD vs. C	rs4149297	0.011	0.3 (0.1–0.7)
*SCARB1*	AD vs. C	rs1031605	0.012	15.8 (1.8–136.2)
*SCARB1*	CRC vs. C	rs7485656	0.021	0.3 (0.1–0.8)
*SCARB1*	CRC vs. AD	rs1031605	0.006	0.2 (0.0–0.6)
*SCARB1*	CRC + AD vs. C	rs7485656	0.012	0.4 (0.2–0.8)
*STARD3*	CRC + AD vs. C	rs11556624	0.04	0.1 (0.0–0.9)
*NPC2*	AD vs. C	rs8008540	0.025	2.5 (1.1–5.6)
*NPC2*	CRC + AD vs. C	rs10139122	0.026	0.4 (0.1–0.9)
*OSBPL5*	AD vs. C	rs11025414	0.012	0.2 (0.1–0.7)
*OSBPL5*	CRC vs. C	rs7122180	0.03	0.1 (0.0–0.8)
*OSBPL5*	CRC vs. AD	rs11025414	0.003	5.5 (1.8–16.6)
*OSBPL5*	CRC + AD vs. C	rs111415444	0.035	0.3 (0.1–0.9)

**Table 2:** Association study results of significant SNPs in genes of “Glycerophospholipid, Sphingolipid metabolism, Glycosylphosphatidylinositol(GPI)-anchor biosynthesis pathway”. AD vs. C, adenoma vs. healthy controls; CRC vs. C, colorectal cancer vs. healthy controls; CRC vs. AD, colorectal cancer vs. adenoma; CRC + AD vs. C, adenomas and colorectal cancer vs. controls. OR, odds-ratio; CI 95%, 95% of confidence interval of odds-ratio.

Gene	Analysis	SNP	*p*	OR (CI 95%)
*LPCAT1*	AD vs. C	rs2962043	0.022	0.3 (0.1–0.8)
*LPCAT1*	CRC vs. C	rs13170616	0.047	0.3 (0.1–1.0)
*LPCAT1*	CRC vs. AD	rs55707452	0.02	14.4 (1.5–136.8)
*LPCAT1*	CRC + AD vs. C	rs2962043	0.018	0.4 (0.2–0.8)
*PLA2G12B*	CRC vs. AD	rs77284811	0.02	0.1 (0.0–0.6)
*PLA2G4C*	AD vs. C	rs1985341	0.016	3.2 (1.2–8.2)
*PLA2G4C*	CRC vs. C	rs8110925	0.006	0.0 (0.0–0.4)
*PLA2G4C*	CRC + AD vs. C	rs8110925	0.005	0.1 (0.0–0.5)
*PLB1*	AD vs. C	rs1528178	0.009	16.6 (2.0–138.4)
*PLB1*	CRC vs. C	rs10176136	0.006	6.7 (1.7–25.9)
*PLB1*	CRC vs. AD	rs7607457	0.001	7.6 (2.3–25.7)
*PLB1*	CRC + AD vs. C	rs80005535	0.014	0.1 (0.0–0.6)
*PLD1*	AD vs. C	rs9823312	0.022	4.2 (1.2–14.2)
*PLD1*	CRC vs. C	rs416158	0.007	0.2 (0.1–0.6)
*PLD1*	CRC vs. AD	rs41273601	0.008	4.1 (1.4–11.4)
*PLD1*	CRC + AD vs. C	rs416158	0.005	0.3 (0.1–0.7)
*PNPLA7*	CRC vs. C	rs12685481	0.033	0.2 (0.0–0.9)
*PNPLA7*	CRC + AD vs. C	rs2488592	0.04	0.4 (0.2–1.0)
*PLPP1*	CRC vs. C	rs111922583	0.019	13.9 (1.6–125.3)
*PLPP1*	CRC + AD vs. C	rs4865950	0.026	0.0 (0.0–0.7)
*PLPP3*	CRC vs. C	rs149181632	0.03	0.1 (0.0–0.8)
*PLPP3*	CRC vs. AD	rs17429642	0.029	0.2 (0.0–0.8)
*CERS4*	AD vs. C	rs7250035	0.017	4.2 (1.3–13.4)
*CERS4*	CRC vs. C	rs11666971	0.01	4.5 (1.4–14.1)
*CERS4*	CRC + AD vs. C	rs7250035	0.022	3.5 (1.2–10.1)
*PIGK*	AD vs. C	rs72683932	0.024	6.4 (1.3–31.7)
*PIGK*	CRC vs. C	rs12562725	0.014	0.2 (0.1–0.7)
*PIGK*	CRC + AD vs. C	rs72683932	0.008	6.5 (1.6–26.5)
*PIGZ*	AD vs. C	rs746037	0.006	3.5 (1.4–8.6)
*PIGZ*	CRC vs. C	rs76347663	0.032	7.9 (1.2–52.3)
*PIGZ*	CRC + AD vs. C	rs746037	0.008	2.7 (1.3–5.8)
*SMPD3*	AD vs. C	rs9940621	0.048	0.3 (0.1–1.0)
*SMPD3*	CRC vs. AD	rs7200932	0.006	0.3 (0.1–0.7)

**Table 3:** Suggestive SNPs associated to microbiome and metabolome traits, which are significant (*p* < 0.05) in adenoma or colorectal cancer. AD vs. C, adenoma vs. healthy controls; CRC vs. C, colorectal cancer vs. healthy controls; CRC vs. AD, colorectal cancer vs. adenoma; CRC + AD vs. C, adenomas and colorectal cancer vs. controls. OR, odds-ratio; CI 95%, 95% of confidence interval of effect or odds-ratio.

			Association with Trait				
Traits	SNP	Gene	*p*	Effect (CI 95%)	Analysis	*p*	OR (CI 95%)
Bacteroidetes	rs2470641	*LOC105376940*	1.7 × 10^−^^6^	−0.086 (−0.119–0.053)	CRC vs. C	0.049	0.3 (0.1–0.9)
Firmicutes	rs2662642	*SPAG16*	1.1 × 10^−^^7^	0.072 (0.048–0.097)	CRC vs. AD	0.034	2.2 (1.1–4.5)
Firmicutes	rs13280938	*LINC01605*	1.6 × 10^−6^	0.14 (0.087–0.194)	CRC vs. AD	0.034	6.8 (1.2–39.8)
Firmicutes	rs9538330	-	4.6 × 10^−6^	−0.073 (−0.103–0.044)	AD vs. C	0.025	3.5 (1.2–10.6)
Firmicutes	rs7166734	*LOC107983974*	2.5 × 10^−6^	−0.077 (−0.107–0.047)	AD vs. C	0.0389	2.7 (1.1–6.9)
Shannon	rs73523611	*CRAT37*	7.66 × 10^−7^	0.42 (0.26–0.57)	AD vs. C	0.044	3.5 (1.0–11.9)
ChoE(18:1)	rs142370545	-	1.97 × 10^−6^	−3.1 (−4.4–1.9)	CRC + AD vs. C	0.049	0.1 (0.01–0.9)
ChoE(18:1)	rs691563	*SLC39A12*	1.14 × 10^−6^	1.0 (0.6–1.3)	CRC vs. C	0.010	5.5 (1.5–20.4)
ChoE(18:1)	rs691563	*SLC39A12*	1.14 × 10^−6^	1.0 (0.6–1.3)	CRC + AD vs. C	0.022	3.4 (1.2–10.2)
ChoE(18:1)	rs6085078	*PROKR2*	1.73 × 10^−6^	0.69 (0.42–0.96)	CRC vs. C	0.021	2.9 (1.2–7.2)
ChoE(18:1)	rs6085078	*PROKR2*	1.73 × 10^−6^	0.69 (0.42–0.96)	CRC + AD vs. C	0.037	2.0 (1.0–3.8)
ChoE(18:2)	rs62249239	*LOC100130207*	1.17 × 10^−6^	1.7 (1.0–2.3)	CRC vs. AD	0.036	9.8 (1.1–83.6)
ChoE(20:4)	rs2146594	*LOC102724520*	4.36 × 10^−6^	−1.1 (−1.6–0.8)	CRC vs. C	0.007	0.2 (0.1–0.7)
ChoE(20:4)	rs56191847	*PCDH15*	3.24 × 10^−6^	−1.4 (−2.0–0.9)	CRC vs. C	0.020	0.3 (0.1–0.8)
ChoE(20:4)	rs56191847	*PCDH15*	3.24 × 10^−6^	−1.4 (−2.0–0.9)	CRC + AD vs. C	0.032	0.4 (0.2–0.9)
PE(16:0/18:1)	rs145994977	-	6.89 × 10^−7^	−3.5 (−4.8–2.2)	CRC + AD vs. C	0.035	0.1 (0.005–0.8)
PE(16:0/18:1)	rs17450393	*SNX2*	1.33 × 10^−6^	−3.1 (−4.3–1.9)	CRC + AD vs. C	0.036	0.1 (0.01–0.8)
PE(16:0/18:1)	rs2048236	*EPM2A*	3.21 × 10^−6^	−1.7 (−2.4–1.1)	AD vs. C	0.027	0.09 (0.01–0.8)
PE(16:0/18:1)	rs2048236	*EPM2A*	3.21 × 10^−6^	−1.7 (−2.4–1.1)	CRC vs. C	0.030	0.1 (0.004–0.8)
PE(16:0/18:1)	rs2048236	*EPM2A*	3.21 × 10^−6^	−1.7 (−2.4–1.1)	CRC + AD vs. C	0.005	0.1 (0.01–0.4)
PE(16:0/18:1)	rs73153821	*CREB3L2*	3.31 × 10^−6^	−2.4 (−3.4–1.5)	CRC + AD vs. C	0.022	0.1 (0.01–0.7)
SM(42:3)	rs17018424	*CCSER1*	2.28 × 10^−6^	−1.7 (−2.4–1.1)	CRC vs. AD	0.012	0.3 (0.1–0.8)
SM(42:3)	rs186239	-	1.13 × 10^−6^	1.7 (1.1–2.4)	CRC vs. C	0.028	5.1 (1.2–21.6)
SM(42:3)	rs186239	-	1.13 × 10^−6^	1.7 (1.1–2.4)	CRC vs. AD	0.041	2.7 (1.0–7.2)
SM(42:3)	rs4745374	*CARNMT1*	2.77 × 10^−6^	1.5 (0.9–2.2)	CRC vs. C	0.014	3.4 (1.3–9.3)
SM(42:3)	rs4745374	*CARNMT1*	2.77 × 10^−6^	1.5 (0.9–2.2)	CRC vs. AD	0.015	3.1 (1.2–7.6)
SM(42:3)	rs11058736	-	1.53 × 10^−6^	2.4 (1.4–3.3)	CRC vs. C	0.030	5.1 (1.2–21.9)
SM(42:3)	rs11058736	-	1.53 × 10^−6^	2.4 (1.4–3.3)	CRC vs. AD	0.014	7 (1.5–33.1)
SM(42:3)	rs6085078	*PROKR2*	2.32 × 10^−6^	1.3 (0.8–1.7)	CRC vs. C	0.021	2.9 (1.2–7.2)
SM(42:3)	rs6085078	*PROKR2*	2.32 × 10^−6^	1.3 (0.8–1.7)	CRC + AD vs. C	0.037	2.0 (1.0–3.8)
SM(42:3)	rs118183318	-	1.75 × 10^−6^	−2.5 (−3.5–1.5)	CRC vs. C	0.012	0.03 (0.002–0.5)
SM(42:3)	rs118183318	-	1.75 × 10^−6^	−2.5 (−3.5–1.5)	CRC vs. AD	0.027	0.1 (0.01–0.7)
SM(42:3)	rs118183318	-	1.75 × 10^−6^	−2.5 (−3.5–1.5)	CRC + AD vs. C	0.032	0.3 (0.1–0.9)
TG(54:1)	rs117956865	*EPS15L1*	2.73 × 10^−8^	−3.1 (−4.1–2.1)	CRC vs. AD	0.039	12.1 (1.1–127.7)

**Table 4:** Results of the area under curve (AUC) of the receiver operating characteristic (ROC) curve in the different models tested. AD vs. C, adenoma vs. healthy controls; CRC vs. C, colorectal cancer vs. healthy controls; CRC vs. AD, colorectal cancer vs. adenoma; CRC + AD vs. C, adenomas and colorectal cancer vs. controls. Model 1, sex, age, metabolome and microbiome data as predictive values; Model 2, sex, age and genetic data (polygenic score and PC1–PC4 components) as predictive values; f-Hb, fecal hemoglobin test as predictive value.

Model	Measurement	AD vs. C	CRC vs. C	AD vs. CRC	CRC + AD vs. C
Model 1	AUC	0.91 (0.84–0.94)	0.87 (0.79–0.91)	0.76 (0.66–0.81)	0.91 (0.86–0.94)
	Specificity	0.81 (0.67–1)	0.92 (0.81–1)	0.8 (0.66–0.91)	0.94 (0.69–1)
	Sensitivity	0.94 (0.64–1)	0.83 (0.69–0.94)	0.72 (0.58–0.86)	0.79 (0.65–0.97)
Model 1 + f-Hb	AUC	1	1	0.77 (0.67–0.82)	0.81 (0.73–0.85)
	Specificity	1	1	0.66 (0.49–0.8)	0.72 (0.58–0.86)
	Sensitivity	1	1	0.89 (0.78–0.97)	0.9 (0.83–0.97)
Model 2	AUC	0.85 (0.76–0.89)	0.85 (0.76–0.89)	0.67 (0.55–0.73)	0.84 (0.76–0.88)
	Specificity	0.82 (0.61–0.92)	0.82 (0.61–0.97)	0.7 (0.32–0.92)	0.79 (0.61–0.95)
	Sensitivity	0.86 (0.7–0.97)	0.82 (0.6–0.98)	0.7 (0.35–0.97)	0.84 (0.65–0.96)
Model 2 + f-Hb	AUC	0.92 (0.86–0.95)	0.95 (0.9–0.98)	0.75 (0.64–0.81)	0.94 (0.89–0.96)
	Specificity	0.82 (0.68–0.92)	0.95 (0.82–1)	0.8 (0.65–0.98)	0.84 (0.68–1)
	Sensitivity	0.97 (0.89–1)	0.9 (0.78–1)	0.7 (0.43–0.86)	0.94 (0.7–1)
Model 1 + Model 2	AUC	0.97 (0.94–0.99)	1	1	0.96 (0.93–0.98)
	Specificity	0.97 (0.83–1)	1	1	1 (0.89–1)
	Sensitivity	0.89 (0.78–1)	1	1	0.86 (0.77–0.97)
Model 1 + Model 2 + f-Hb	AUC	1	1	1	0.93 (0.87–0.96)
	Specificity	1	1	1	0.89 (0.78–0.97)
	Sensitivity	1	1	1	0.97 (0.93–1)

## Data Availability

The data presented in this study are available on request from the authors. The data are not publicly available due to ethical reasons (genotype data cannot be shared).

## Supplementary Materials

The following are available online at https://www.mdpi.com/article/10.3390/cancers13246216/s1, Supplementary Table S1: Association study results of SNP previously reported to be associated with colorectal cancer.
