# Supplementary material for: Interplay between Genome, Metabolome and Microbiome in Colorectal Cancer
Source: Cancers (Basel). 2021 Dec 10;13(24):6216. doi: 10.3390/cancers13246216 (PMC8699218; doi:10.3390/cancers13246216)
Supplement: Supplementary file 1 [file cancers-13-06216-s001.zip › cancers-1480935-supplementary.pdf]

Interplay between genome, metabolome and microbiome in colorectal cancer

Koldo Garcia-Etxebarria, Marc Clos-Garcia, Oiana Telleria, Beatriz Nafría, Cristina Alonso, Marta Iruarrizaga-Lejarreta, Andre Franke, Anais Crespo, Agueda Iglesias, Joaquín Cubiella, Luis Bujanda and Juan Manuel Falcón-Pérez

**Supplementary material**	**Page**
Supplementary Table S1	2

Supplementary Table S1: Association study results of SNP previously reported to be associated with colorectal cancer. AD vs C, adenoma vs healthy controls; CRC vs C, colorectal cancer vs healthy controls; CRC vs AD, colorectal cancer vs adenoma; CRC+AD vs C, adenomas and colorectal cancer vs controls. OR, odds-ratio; CI 95%, 95 % of confidence interval of odds-ratio.

	**AD vs C**		**CRC vs C**		**CRC vs AD**		**CRC+AD vs C**	
**SNP**	**P**	**OR (CI 95%)**	**P**	**OR (CI 95%)**	**P**	**OR (CI 95%)**	**P**	**OR (CI 95%)**
rs4500715	0.655	1.3 (0.4-4.5)	0.358	0.4 (0.1-2.5)	0.13	0.4 (0.1-1.4)	0.65	0.8 (0.2-2.4)
rs61776719	0.968	1.0 (0.4-2.6)	0.095	2.2 (0.9-5.6)	0.189	1.6 (0.8-3.4)	0.402	1.4 (0.7-2.9)
rs12143541	0.282	0.5 (0.1-1.7)	0.954	1.0 (0.3-3.2)	0.265	1.9 (0.6-6.1)	0.389	0.6 (0.2-1.8)
rs11893063	0.79	0.9 (0.4-1.9)	0.583	1.2 (0.6-2.6)	0.98	1.0 (0.5-2.1)	0.927	1.0 (0.5-1.9)
rs7593422	0.12	1.7 (0.9-3.5)	0.437	1.4 (0.6-3.1)	0.646	0.9 (0.4-1.7)	0.143	1.6 (0.9-3.0)
rs9831861	0.376	0.7 (0.3-1.6)	0.09	0.5 (0.2-1.1)	0.649	0.8 (0.4-1.8)	0.131	0.6 (0.3-1.2)
rs12635946	0.397	0.7 (0.3-1.7)	0.162	1.9 (0.8-5.0)	0.618	1.2 (0.6-2.7)	0.271	1.5 (0.7-3.1)
rs17035289	0.231	0.5 (0.2-1.5)	**0.017**	0.2 (0.1-0.8)	0.284	0.6 (0.2-1.6)	0.086	0.4 (0.2-1.1)
rs75686861	0.104	2.8 (0.8-9.6)	0.594	1.5 (0.3-7.4)	0.338	0.6 (0.2-1.8)	0.115	2.5 (0.8-8.0)
rs3131043	0.695	1.2 (0.5-2.6)	0.297	1.6 (0.7-3.6)	0.364	1.4 (0.7-3.1)	0.454	1.3 (0.7-2.5)
rs9271770	0.859	1.1 (0.4-3.1)	0.86	0.9 (0.3-2.8)	0.729	0.8 (0.3-2.5)	0.747	0.9 (0.4-2.1)
rs3801081	0.988	1.0 (0.4-2.3)	0.993	1.0 (0.4-2.4)	0.587	0.8 (0.3-1.9)	0.943	1.0 (0.5-2.0)
rs1412834	0.426	0.7 (0.3-1.6)	0.193	0.6 (0.3-1.3)	0.793	0.9 (0.4-2.0)	0.137	0.6 (0.3-1.2)
rs12427600	0.61	1.3 (0.5-3.1)	0.872	1.1 (0.4-2.8)	0.368	0.7 (0.3-1.6)	0.805	1.1 (0.5-2.4)
rs7993934	0.094	0.4 (0.2-1.1)	0.626	1.3 (0.5-3.2)	0.177	1.8 (0.8-4.5)	0.296	0.7 (0.3-1.4)
rs4776316	0.437	0.7 (0.3-1.7)	0.847	0.9 (0.4-2.2)	0.359	1.5 (0.6-3.6)	0.481	0.8 (0.4-1.6)
rs7495132	0.818	1.2 (0.3-5.2)	0.398	2.0 (0.4-9.2)	0.737	1.2 (0.3-4.6)	0.673	1.3 (0.4-4.9)
rs61336918	0.312	0.7 (0.3-1.4)	0.177	0.6 (0.3-1.3)	0.986	1.0 (0.4-2.3)	0.133	0.6 (0.3-1.2)
rs285245	0.832	0.9 (0.2-3.7)	0.374	1.7 (0.5-5.5)	0.296	1.8 (0.6-5.8)	0.662	1.3 (0.4-3.7)
rs12979278	0.972	1.0 (0.4-2.2)	0.786	0.9 (0.4-1.9)	0.742	1.1 (0.6-2.3)	0.781	0.9 (0.5-1.8)
rs3787089	0.665	1.2 (0.5-3.1)	**0.022**	3.1 (1.2-8.1)	**0.01**	2.8 (1.3-6.1)	0.1	1.9 (0.9-4.1)
rs6983267	0.594	1.2 (0.6-2.6)	0.297	0.6 (0.3-1.5)	0.08	0.5 (0.2-1.1)	0.758	0.9 (0.5-1.8)
rs16892766	0.573	1.5 (0.4-6.1)	0.508	0.6 (0.1-3.0)	0.371	0.6 (0.2-1.9)	0.872	1.1 (0.3-4.1)
rs10795668	0.117	2.0 (0.8-4.5)	0.403	1.5 (0.6-3.8)	0.461	0.8 (0.4-1.6)	0.147	1.8 (0.8-3.8)
rs3802842	0.83	1.1 (0.4-3.1)	0.811	0.9 (0.3-2.4)	0.695	1.2 (0.5-2.9)	0.619	1.2 (0.5-2.9)
rs4779584	0.559	1.4 (0.5-4.2)	0.209	2.2 (0.6-7.3)	0.631	1.3 (0.5-3.4)	0.33	1.6 (0.6-4.3)
rs4444235	0.071	2.1 (0.9-4.7)	0.73	1.2 (0.5-2.6)	0.577	0.8 (0.4-1.7)	0.147	1.7 (0.8-3.3)
rs9929218	0.974	1.0 (0.4-2.2)	0.307	0.6 (0.3-1.5)	0.256	0.6 (0.3-1.4)	0.527	0.8 (0.4-1.6)
rs10411210	0.271	1.8 (0.6-4.9)	0.758	0.8 (0.3-2.6)	0.054	0.4 (0.1-1.0)	0.582	1.3 (0.5-3.1)
rs961253	0.874	0.9 (0.4-2.0)	0.548	1.3 (0.6-2.8)	0.468	1.3 (0.6-2.9)	0.96	1.0 (0.5-1.9)
rs1801133	**0.015**	0.3 (0.1-0.8)	**0.01**	0.4 (0.2-0.8)	0.792	0.9 (0.4-1.9)	**0.003**	0.4 (0.2-0.7)
rs1801282	0.109	0.3 (0.1-1.3)	0.273	0.5 (0.1-1.9)	0.426	1.9 (0.4-9.3)	0.123	0.4 (0.1-1.3)
rs4073	0.102	2.2 (0.9-5.5)	0.282	1.6 (0.7-3.9)	0.903	1.0 (0.5-2.2)	0.1	1.9 (0.9-4.0)
rs1800795	0.362	1.5 (0.6-3.8)	0.123	2.1 (0.8-5.5)	0.648	1.2 (0.5-2.6)	0.153	1.8 (0.8-3.8)
rs9344	0.729	1.1 (0.6-2.3)	0.277	1.5 (0.7-3.4)	0.089	2.0 (0.9-4.2)	0.323	1.4 (0.7-2.6)
rs10067	0.617	0.7 (0.2-2.6)	0.567	0.7 (0.2-2.4)	0.965	1.0 (0.3-3.9)	0.87	0.9 (0.3-2.6)
rs2102302	0.303	0.7 (0.3-1.4)	0.794	0.9 (0.4-2.0)	0.418	1.4 (0.7-2.8)	0.535	0.8 (0.4-1.6)
rs12732	0.898	0.9 (0.3-2.5)	0.455	1.5 (0.5-4.5)	0.641	1.2 (0.5-3.0)	0.688	1.2 (0.5-2.8)
rs4679392	0.33	1.5 (0.7-3.2)	0.441	0.7 (0.3-1.6)	0.062	0.5 (0.2-1.0)	0.906	1.0 (0.5-1.8)
rs6826961	0.136	2.3 (0.8-6.5)	0.169	2.1 (0.7-5.9)	0.767	1.1 (0.5-2.7)	0.162	1.9 (0.8-4.4)
rs6580076	0.368	1.6 (0.6-4.6)	0.475	0.6 (0.2-2.2)	0.094	0.4 (0.1-1.2)	0.865	1.1 (0.4-2.7)
rs2277937	**0.037**	0.3 (0.1-0.9)	0.14	0.5 (0.2-1.2)	0.188	1.9 (0.7-4.9)	0.062	0.5 (0.2-1.0)
rs1634730	0.547	1.4 (0.5-3.6)	0.98	1.0 (0.3-3.2)	0.448	0.7 (0.2-1.8)	0.928	1.0 (0.4-2.5)
rs11766125	0.932	1.0 (0.4-2.1)	0.463	0.7 (0.3-1.9)	0.706	0.8 (0.4-2.0)	0.525	0.8 (0.4-1.7)
rs15783	0.875	0.9 (0.4-2.0)	0.423	1.4 (0.6-3.2)	0.399	1.4 (0.7-2.8)	0.706	1.1 (0.6-2.2)
rs11029621	0.516	1.3 (0.6-2.7)	0.232	1.7 (0.7-3.8)	0.542	1.3 (0.6-2.7)	0.268	1.5 (0.8-2.8)
rs2933353	0.764	0.9 (0.4-2.1)	0.426	0.7 (0.3-1.6)	0.892	0.9 (0.4-2.1)	0.745	0.9 (0.4-1.9)
rs2230283	0.772	1.1 (0.5-2.3)	0.479	1.3 (0.6-2.9)	0.817	1.1 (0.5-2.1)	0.681	1.1 (0.6-2.1)
rs17647532	0.991	1.0 (0.4-2.4)	0.485	0.7 (0.2-2.0)	0.23	0.6 (0.2-1.4)	0.547	0.8 (0.3-1.7)
rs1862458	0.136	0.5 (0.2-1.2)	0.63	0.8 (0.3-2.0)	0.157	1.9 (0.8-4.4)	0.224	0.6 (0.3-1.3)
rs10752881	0.383	1.9 (0.4-8.4)	0.257	2.2 (0.6-8.8)	0.295	1.4 (0.7-2.9)	0.301	1.9 (0.6-6.1)
